# Personality and individual attitudes toward vaccination: a nationally representative survey in the United States

**DOI:** 10.1186/s12889-020-09840-w

**Published:** 2020-11-23

**Authors:** Fang-Yu Lin, Ching-Hsing Wang

**Affiliations:** 1 Department of Symptom Research, The University of Texas M.D. Anderson Cancer Center, 1515 Holcombe Boulevard, Unit 1450, Houston, TX 77030 USA; 2 Department of Political Science, National Cheng Kung University, No.1, University Road, Tainan City, 701 Taiwan, Republic of China

**Keywords:** Vaccination, The big five, Personality, Vaccination attitude

## Abstract

**Background:**

Although past studies have identified factors associated with individual perceptions of vaccination, limited attention has been paid to the role of personality in individual attitudes toward vaccination. This study aimed to evaluate the effect of personality as measured by the Big Five personality traits on individual attitudes toward vaccination using a nationally representative survey in the United States.

**Methods:**

A cross-sectional study was conducted with a sample of 3276 American citizens who were aged 18 and above and lived in 50 U.S. states and Washington D.C. from the American National Election Studies. The survey was collected through face-to-face and online interviews using structured questionnaires in 2016. The multistage stratified cluster sampling procedure was used for face-to-face interview, whereas the USPS DSF was used to select the sample for online interview. Multivariable ordinal logistic regression was used to assess how personality traits (extraversion, agreeableness, conscientiousness, emotional stability, and openness to experience) as main explanatory variables influence the outcome variables – individual attitudes toward health benefits of vaccination and support for school vaccination.

**Results:**

More than two-thirds of respondents perceive health benefit of vaccination and support vaccination requirements for school entry, whereas about one-tenth of respondents have safety concerns about vaccination and oppose the vaccination requirements. After adjusting for ideology, insurance status, and demographic variables, the traits of agreeableness, conscientiousness and emotional stability remain significantly associated with attitude toward vaccination; conscientiousness is significantly associated with support for school vaccination. The odds of reporting health benefits of vaccination associated with one-point increase in agreeableness, conscientiousness and emotional stability are 1.05 (95% confidence intervals [CI] = 1.01–1.08), 1.05 (95% CI = 1.02–1.09) and 1.03 (95% CI = 1.00–1.06), respectively. For a one-point increase in conscientiousness, the odds of supporting school vaccination increase by 1.08 (95% CI = 1.05–1.12).

**Conclusions:**

People high in agreeableness, conscientiousness and emotional stability are more likely to regard vaccination as beneficial, whereas those high in conscientiousness are more likely to support school-based vaccine requirement. This study highlights the importance of personality in shaping individual attitudes toward vaccination. More research is needed to understand the role of personality in individual health attitudes and behavior.

## Background

Vaccination is one of the most effective approaches to protect individuals from serious illness and complications of vaccine-preventable diseases. It has been observed that anti-vaccination groups have fought back against public health campaigns over the past years. In 2019, the World Health Organization (WHO) listed the anti-vaccination movements as one of ten biggest health threats to endanger global health [1]. Although state laws have established vaccination requirements for school entry, exemptions are still allowed. Parents can refuse to have their children vaccinated due to medical reasons, religious reasons, philosophical or personal beliefs [2]. Minority groups such as Blacks and Hispanics [3], people without health insurance [4], and those with low levels of household income [5] are less likely to get vaccinated. Unvaccinated children not only tend to have a higher risk of vaccine-preventable diseases, but also pose a substantial economic burden. According to estimates from the Centers for Disease Control and Prevention, routine vaccination among children born 1994–2018 will prevent an estimated 419 million illnesses, 26.8 million hospitalizations, and 936,000 early deaths over the course of their lifetimes, at a net savings of $406 billion in direct costs and $1.9 trillion in total societal costs [6]. As vaccine refusal for nonmedical reasons is a growing concern in the United States, there is a pressing need to find motivated reasoning perspectives to overcome fears and myths about vaccination, and thus design effective intervention to improve vaccine uptake.

While previous research on predictors of non-vaccination has focused on specific beliefs about vaccine, or the demographic and social characteristics, very few scholarly attention has been paid to the role of dispositional factors in explaining individual attitudes toward vaccination. Personality represents the set of dispositional traits, thinking patterns and habitual behaviors within individuals and influences how individuals respond to external stimuli and interact with other people in a society. It has been widely recognized that the five-factor model of personality, often known as the Big Five, covers broader personality dimensions and has the advantage to examine the individual or interactive effects of personality traits on health behaviors. The personality characteristics can be organized in terms of five domains: extroversion (i.e., being energetic, outgoing and sociable), agreeableness (i.e., trustworthy, altruistic and sympathetic), conscientiousness (i.e., self-disciplined, dutiful and thoughtful), emotional stability (i.e., calm, relaxed and even-tempered), and openness to experience (i.e., creative, curious and open to new ideas) [7].

Previous research has documented that the Big Five personality traits exert considerable influence on health behaviors, physical activity and dietary habit [8, 9], but less effort has been put forth on vaccination attitudes. Although a cross-sectional study attempted to uncover the psychological roots of anti-vaccinations attitudes, it simply focused on how conspiratorial beliefs, disgust, reactance, and individualism/hierarchical worldviews influenced anti-vaccinations attitudes without talking about personality [10]. So far only one study examined the correlations between personality traits and individual attitudes toward the safety of childhood vaccinations in New Zealand [11]. It remains unclear whether personality traits could come into play in terms of individual attitudes toward vaccination in the United States. To fill this gap, this study employed a nationally representative sample to investigate the effects of personality traits on individual attitudes toward vaccination, especially focusing on perception of benefit and risk of vaccination and support for school vaccination.

## Methods

This cross-sectional study utilized data from the 2016 American National Election Studies (ANES) Time Series Study. The ANES Time Series Study is a publicly available data set that included pre-election and post-election surveys collected through face-to-face and online interviews using structured questionnaires. Pre-election interviews were conducted with study respondents during the 2 months prior to the 2016 elections and were followed by post-election reinterviewing beginning November 9, 2016. Since all variables used in this study were obtained from the post-election survey, this study only used data from the post-election survey for empirical analysis.

### Study sample

Two independently probability samples were drawn respectively for face-to-face and online interviews. The target population was American citizens aged 18 and above in 50 U.S. states and Washington D.C. except that Alaska and Hawaii were not included in the face-to-face interview. The lists of residential addresses were used as the sampling frame for both interviews. The multistage stratified cluster sampling procedure was used for face-to-face interview. First, random probability proportion of adult citizens was selected in the 60 primary sampling units (PSU). Then four smaller areas were drawn within each PSU, and finally households were selected at random from the U.S. Postal Service’s computerized delivery sequence file (USPS DSF). Besides, the USPS DSF was used to randomly select the sample for online interview. The respondents were recruited by mail and questionnaires were administered on the Internet.

The sample consisted of 3648 respondents. 1058 respondents were interviewed face-to-face, whereas the rest of the respondents were interviewed online. Given missing values due to nonresponses, the effective number of observations for empirical analysis was reduced to 3276 in this study. In addition, sample weights were constructed respectively for the internet sample, the face-to-face sample, and the two samples combined to make valid inferences about the population. Since this study used the combined sample to perform empirical analysis, composite weights for the combined sample based on the raking procedure that included the dimensions of age by gender, ethnicity by educational attainment, marital status by gender, ethnicity by census region, nation of birth and home tenure by metropolitan status were utilized to produce estimates of the relationships between personality traits and individual attitudes toward vaccination.

### Measures

Demographic data were collected on age, gender, ethnicity, educational attainment, marital status, family income, ideology and insurance status. Ideology was assessed with an eight-point scale ranging from 1 to 7, and a higher value indicates that an individual was more liberal. In terms of insurance status, it was dichotomously categorized to have health insurance and no health insurance. The primary predictor of interest was the Big Five personality traits as measured by the Ten-Item Personality Inventory (TIPI) developed by Gosling, Rentfrow, and Swann [12]. The TIPI was previously validated with high validity and provided reliable assessment of personality. It has been commonly used to assess personality for research settings in which the respondents have limited time to respond to the survey due to its brevity in terms of the number of questions. Each personality trait was measured by two items and the score for each personality trait was obtained by adding, after appropriate recoding, the two items for each particular personality dimension. A higher score signified that an individual had a more prominent personality trait and each personality trait was coded to range from 2 to 14.

The primary outcome of the study was individual attitudes toward vaccination as assessed by the four survey questions. Two questions asked the respondents to express their perceptions of benefit and risk of vaccination. Specifically, the respondents first answered whether health benefits of vaccinations generally outweighed the risks, risks outweighed the benefits, or there was no difference. Then they identified intensity levels of their opinions using a three-point scale. Therefore, individual attitude toward health benefits of vaccination was assessed on a seven-point scale by combining the respondents’ answers to these two questions. Similarly, two questions asked the respondents to express their support for school vaccination. The respondents first answered whether they favored, opposed, or neither favored nor opposed requiring children to be vaccinated in order to attend public schools. Then they identified intensity levels of their opinions using a three-point scale. Thus, individual support for school vaccination was assessed on a seven-point scale by combining the respondents’ answers to these two questions. The ANES questionnaire offered valid and reliable measurement of individual attitudes and behavior. To ensure the validity of the survey, ANES staff periodically monitored each interviewer and reviewed the data during the interviewing period. Separate pretests were conducted before the interview and demonstrated good validity.

### Statistical analysis

This study treated individual attitudes toward vaccinations as ordinal variables. The higher values indicated that the respondents regarded vaccination as more beneficial or were more supportive of school vaccination. Ordinal logistic regression was used to estimate cross-sectional associations between personality traits and individual attitudes toward vaccination, adjusting for potential confounders based on a priori knowledge, including gender, age, ethnicity, education attainment, family income, insurance status, and ideology. All the analyses were performed with Stata, version 16.0, during the year 2019 and all *p*-values were two-sided and considered statistically significant at an α level of 0.05.

## Results

Table 1 demonstrates the characteristics of the respondents (*n* = 3276). In general, the majority (72%) of the respondents viewed vaccination as beneficial and slightly more than one-tenth (12%) of the respondents were worried about the risk of vaccination. Similarly, the majority (77%) of the respondents supported the requirement of vaccination for children to attend public schools, whereas less than one-tenth (8%) of the respondents opposed this requirement. Table 2 shows the results for the correlations between personality traits and individual attitudes toward vaccination. Personality traits are significantly associated with individual attitudes toward vaccination. Specifically, the traits of agreeableness, conscientiousness, emotional stability and openness to experience are significantly positively correlated with attitude toward health benefits of vaccination. By contrast, only two traits – agreeableness and conscientiousness – are significantly positively associated with support for school vaccination.

**Table 1:** Participant characteristics (*n* = 3276)

Characteristics	Mean (SD)	Frequency (%)
Age	46.9 (17.6)	
Sex		
Men		49
Women		51
Race		
White		70
Black		11
Hispanic		12
Others		7
Income	15.8 (8.1)	
Years of education	10.7 (2.4)	
Insurance status		
Yes		90
No		10
Liberal ideology	3.9 (1.5)	
Personality traits		
Extraversion	8.4 (2.8)	
Agreeableness	10.3 (2.3)	
Conscientiousness	11.3 (2.3)	
Emotional stability	9.8 (2.6)	
Openness to experience	10.0 (2.2)	
Support for school vaccination		
Oppose a great deal		4
Oppose a moderate amount		3
Oppose a little		1
Neither favor nor oppose		15
Favor a little		3
Favor a moderate amount		17
Favor a great deal		57
Attitude toward health benefits of vaccination		
Risks much greater		2
Risk moderately greater		7
Risk slightly greater		3
No difference		16
Benefits slightly greater		8
Benefits moderately greater		20
Benefits much greater		44

**Table 2:** Correlations between personality traits and individual attitudes toward vaccination

Variable	Attitude toward health benefits of vaccination		Support for school vaccination	
	*r*	*p*-value	*r*	*p*-value
Extraversion	0.002	0.922	0.015	0.383
Agreeableness	0.158	**< 0.001**	0.088	**< 0.001**
Conscientiousness	0.156	**< 0.001**	0.119	**< 0.001**
Emotional stability	0.119	**< 0.001**	0.017	0.340
Openness to experience	0.065	**< 0.001**	0.008	0.629

Note: Boldface indicates statistical significance (*p* < 0.001)

**Fig. 1:**
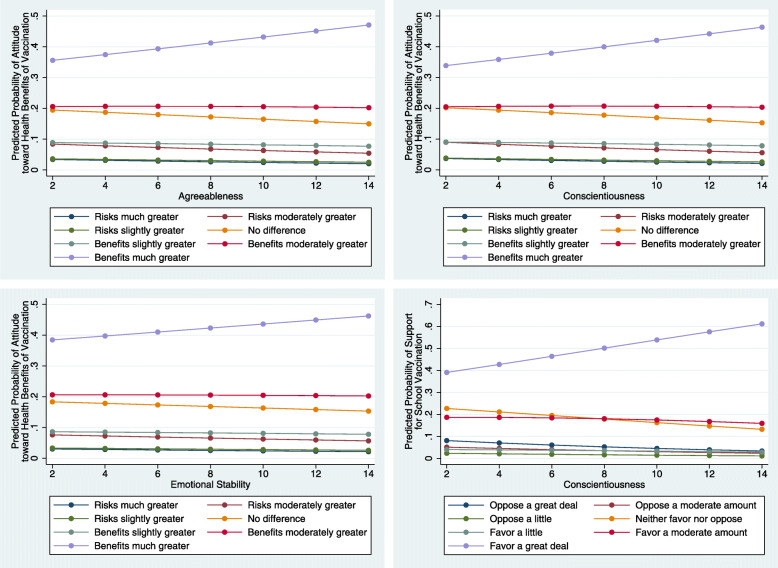
The effects of personality traits on individual attitudes toward vaccination

Table 3 examines the associations of personality traits with individual attitudes toward vaccination after adjusting for demographic variables, insurance status and ideology. After adjustment, agreeableness, conscientiousness and emotional stability are still significantly positively associated with attitude toward health benefits of vaccinations. For a one-point increase in agreeableness, conscientiousness and emotional stability, the odds of reporting health benefits of vaccination increase respectively by a factor of 1.05 (95% confidence intervals [CI] = 1.01–1.08), 1.05 (95% CI = 1.02–1.09) and 1.03 (95% CI = 1.00–1.06), holding all other variables constant. On the other hand, conscientiousness is the only one trait that is significantly positively associated with support for school vaccination. For a one-point increase in conscientiousness, the odds of supporting school vaccination increase by a factor of 1.08 (95% CI = 1.05–1.12), holding all other variables constant. Figure 1 demonstrates the substantive effects of statistically significant personality traits on individual attitudes toward vaccination by calculating predicted probabilities for each of these categories. With the increase of agreeableness, conscientiousness and emotional stability, the predicted probabilities of viewing health benefits of vaccination as much greater respectively increase by 11.5, 12.5 and 7.8%. Furthermore, as conscientiousness increases, the predicted probability of favoring school vaccination a great deal increases by 22.1%.

**Table 3:** Ordered logit analysis of individual attitudes toward vaccination

Variable	Attitude toward health benefits of vaccination			Support for school vaccination		
	Coef.	OR	*p*-value	Coef.	OR	*p*-value
	(SE)	(95% CI)		(S.E.)	(95% CI)	
Extraversion	−0.022	0.98	0.080	0.011	1.01	0.407
	(0.013)	(0.95, 1.00)		(0.013)	(0.99, 1.04)	
Agreeableness	0.045	1.05	0.007**	0.032	1.03	0.065
	(0.017)	(1.01, 1.08)		(0.017)	(1.00, 1.07)	
Conscientiousness	0.049	1.05	0.003**	0.078	1.08	***p*** < 0.001***
	(0.017)	(1.02, 1.09)		(0.017)	(1.05, 1.12)	
Emotional stability	0.030	1.03	0.037*	−0.025	0.97	0.095
	(0.015)	(1.002, 1.06)		(0.015)	(0.95, 1.00)	
Openness to experience	0.010	1.01	0.571	−0.024	0.98	0.175
	(0.017)	(0.98, 1.04)		(0.018)	(0.94, 1.01)	
Liberal ideology	0.163	1.18	***p*** < 0.001***	0.141	1.15	***p*** < 0.001***
	(0.024)	(1.12, 1.23)		(0.025)	(1.10, 1.21)	
Insurance status	0.370	1.45	0.001**	0.318	1.37	0.005**
	(0.109)	(1.17, 1.79)		(0.113)	(1.10, 1.71)	
Income	0.020	1.02	***p*** < 0.001***	0.012	1.01	0.010*
	(0.005)	(1.01, 1.03)		(0.005)	(1.003, 1.02)	
Years of education	0.157	1.17	***p*** < 0.001***	0.039	1.04	0.017*
	(0.016)	(1.13, 1.21)		(0.016)	(1.01, 1.07)	
Race						
Black	−1.072	0.34	***p*** < 0.001***	−0.488	0.61	***p*** < 0.001***
	(0.104)	(0.28, 0.42)		(0.110)	(0.49, 0.76)	
Hispanic	−0.663	0.52	***p*** < 0.001***	−0.169	0.84	0.116
	(0.103)	(0.42, 0.63)		(0.108)	(0.68, 1.04)	
Others	−0.522	0.59	***p*** < 0.001***	0.009	1.01	0.949
	(0.125)	(0.46, 0.76)		(0.133)	(0.78, 1.31)	
Female	0.102	1.11	0.132	0.146	1.16	0.041*
	(0.068)	(0.97, 1.27)		(0.071)	(1.01, 1.33)	
Age	0.011	1.01	***p*** < 0.001***	0.013	1.01	***p*** < 0.001***
	(0.002)	(1.01, 1.01)		(0.002)	(1.01, 1.02)	
N	3276			3276		
Likelihood ratio test	632.23			214.09		
*p*-value	***p*** < 0.001***			***p*** < 0.001***		
Pseudo *R*^*2*^	0.061			0.025		

Note: Boldface indicates statistical significance (*: *p* < 0.05; **: *p* < 0.01; ***: *p* < 0.001)

Besides, ideology, insurance status and such demographic characteristics as income, education, and age are consistently and significantly associated with individual attitudes toward vaccination. Specifically, people who are older or ideologically liberal, or have health insurance or higher levels of income and education are inclined to view vaccination as beneficial and support school vaccination. Compared to whites, all the other ethnic groups tend to regard vaccination as risky, whereas women are more likely than men to support school vaccination.

## Discussion

This study provides concrete evidence on the relationships between personality traits and individual attitudes toward vaccination. Specifically, agreeableness and emotional stability exert significant positive effects on individual attitudes toward health benefits of vaccination, whereas conscientiousness has a significant positive influence on individual attitudes toward health benefits of vaccination and support for school vaccination. That is, people high in agreeableness, conscientiousness and emotional stability are more likely to perceive vaccination as beneficial, whereas those high in conscientiousness are also more likely to favor school-based vaccine requirement.

It seems that the conscientiousness is the most important personality trait to shape individual attitudes toward vaccination. Conscientiousness is characterized by the tendency to show self-discipline and act dutifully and thus people high in conscientiousness tend to abide by social norms [7]. Since substantial research has documented that social norms are associated with vaccination [13, 14], the emphasis of norm abidance would motive conscientious people to be more receptive to vaccination. Besides, in line with previous research showing that people low in conscientiousness are more likely to violate precaution rules [15], our finding suggests that people high in conscientiousness tend to adopt preventive health measures. On the other hand, mixed evidence on the impact of agreeableness and emotional stability indicates that although agreeableness and emotional stability could influence individual perceptions about the benefits and risks of vaccination, they do not come into play in terms of support for mandatory school vaccinations. This suggests that people with higher levels of agreeableness and emotional stability might not think of mandatory school vaccinations as beneficial; otherwise, they should be in favor of such vaccination requirements given significant associations of agreeableness and emotional stability with individual attitudes toward health benefits of vaccination.

Since genetics plays a major role in personality formation [16], it has been assumed that personality traits are causally prior to any specific attitudes or behaviors and are important determinants of a wide range of attitudes and behavior. While our findings demonstrate that individuals’ attitudes toward vaccination are determined by their intrinsic dispositions, our study does not negate the idea that exposure to accurate information about vaccination can positively impact people’s attitudes to vaccination [17]. In fact, past studies have revealed that personality traits are associated with information needs and seeking behavior [18, 19]. When accurate information about vaccination is provided, people with some specific personality traits would be more likely to learn or receive such information and further change their attitudes toward vaccination. Therefore, personality traits and health information provision can interact with each other to form attitudes about vaccination.

In addition to personality, our study finds that ideology, insurance status, income, education and age are consistently and significantly associated with individual perceptions of benefits and risks of vaccination and support for school vaccination. In line with previous research [20], our study reveals that liberals are more likely to hold positive attitudes toward vaccination. This might be because conservatives are more sensitive to threat and more risk averse than those who are liberal. Conservatives are inclined to be vaccine skeptics and less likely to express pro-vaccination attitudes. Besides, the findings on the associations of demographic characteristics with individual attitudes toward vaccination seem to imply that people with high socioeconomic status are more likely to have pro-vaccination attitudes. This might provide a warning signal that the government should make efforts to increase economically disadvantaged individuals’ awareness of vaccination in order to protect them from vaccine preventable diseases.

This is the first study to use a nationally representative sample to examine the relationships between personality traits and individual attitudes toward vaccination. The results may be generalized to the entire adult population in the U.S. Additionally, the study is less prone to selection bias because of the representative study population. However, given data limitations, this study could simply address the effects of personality traits on individual attitudes toward health benefits of vaccination and support for school vaccination. People might have differentiated attitudes toward the benefits and risks of different types of vaccines and either support or oppose different vaccination policies. Therefore, future research can be done to examine the relationships between personality traits and individual attitudes toward vaccination more specifically. Besides, there might exist mediation relationships between personality traits and individual attitudes toward vaccination. Nonetheless, given lack of appropriate mediators, this study could not examine such a mediation mechanism and calls for further investigation into the identification of mediators for the relationships between personality traits and individual attitudes toward vaccination.

## Conclusions

This study used a nationally representative sample of adults in the United States to examine the relationships between the Big Five personality traits and individual attitudes toward vaccination, showing that some personality traits have significant effects on individual attitudes toward vaccination. This highlights the importance of dispositional characteristics in shaping individual health attitudes and behavior. Therefore, when it comes to the factors affecting individual health attitudes and behavior, we should take the role of personality into consideration as well. Finally, given that there exist significant differences in geographic distribution of the Big Five personality traits, more research is needed to determine if the findings of this study can be generalized to other countries.

## Data Availability

The datasets analyzed during the current study are in the public domain and are freely available from https://electionstudies.org/.

## Abbreviations

ANES: American National Election Studies
CI: Confidence Interval
OR: Odds Ratio
TIPI: Ten-Item Personality Inventory
WHO: World Health Organization
